# ExoCarta as a resource for exosomal research

**DOI:** 10.3402/jev.v1i0.18374

**Published:** 2012-04-16

**Authors:** Richard J. Simpson, Hina Kalra, Suresh Mathivanan

**Affiliations:** Department of Biochemistry, La Trobe Institute for Molecular Science, La Trobe University, Melbourne, Victoria, Australia

**Keywords:** exosome resource, microvesicle database, exosome tools, extracellular organelles, Vesiclepedia

## Abstract

Exosomes are a class of extracellular vesicles that are secreted by various cell types. Unlike other extracellular vesicles (ectosomes and apoptotic blebs), exosomes are of endocytic origin. The roles of exosomes in vaccine/drug delivery, intercellular communication and as a possible source of disease biomarkers have sparked immense interest in them, resulting in a plethora of studies. Whilst multidimensional datasets are continuously generated, it is difficult to harness the true potential of the data until they are compiled and made accessible to the biomedical researchers. Here, we describe ExoCarta (http://www.exocarta.org), a manually curated database of exosomal proteins, RNA and lipids. Datasets currently present in ExoCarta are integrated from both published and unpublished exosomal studies. Since its launch in 2009, ExoCarta has been accessed by more than 16,000 unique users. In this article, we discuss the utility of ExoCarta for exosomal research and urge biomedical researchers in the field to deposit their datasets directly to ExoCarta.

Exosomes, membranous vesicles of endocytic origin, are signalling organelles secreted by normal and disease cells (1–5). Originally described three decades ago (6, 7), exosomes contain a subproteome of the cells and are found in many bodily fluids (1, 8). Released upon fusion of multivesicular bodies (MVBs) with the plasma membrane (PM), exosomes are of 40–100 nm in diameter, are of endocytic origin, have a cup shaped appearance as visioned by electron microscopy, have a buoyant density in sucrose of 1.10–1.21 g/mL and sediment at 100,000 *g* (9, 10). They harbour proteins/RNA/lipids that reflect the functionality of the host cell and posses molecular signatures or footprints resembling the diseased cell from which they were secreted (11). Exosomes exhibit a typical lipid bilayer membrane and are high in phosphatidylserine (PS) residues on their surface (10). The field of exosomes has witnessed renewed interest in the past 5 years mainly due to the discovery of luminal RNAs including mRNA and miRNA in exosomes (12). The finding of exosome mediated non-selective transfer (12) of inactive forms of both mRNA and miRNAs to neighbouring cells has spurred numerous studies on exosomes (Fig. 1). This enormous interest in exosomal studies can be attributed to 3 main reasons: (1) purported role of exosomes in intercellular signalling; (2) use as delivery vehicles for vaccines and drugs and (3) as possible sources of disease biomarkers.

**Fig. 1:**
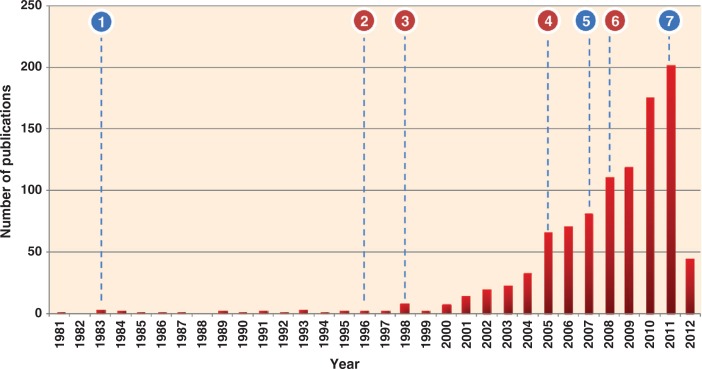
Histogram of exosomal studies over the past 30 years. Immense interest in exosomes was seen during the last 7 years; more than 70% of the studies on exosomes were published (2005–2012). The statistics is generated based on PubMed indexed exosomal studies (keywords: exosomes or exosome-like) and adding the initial observations on exosomes. *The resulting articles were manually verified by reading the title and abstract to ensure that the study referred exosomes, secreted vesicles of endocytic origin*. Whilst each exosomal article improved our biological understanding, several articles contributed significantly. Important discoveries reported in the last 30 years are highlighted in the figure (blue – these findings moved exosomal research to a new next level; pink – trendsetting studies that formed the basis of current exosomal research): −1, discovery of exosomes (6, 47); −2, immune functions of B cell-secreted exosomes (48); −3, exosomes promote induction of antitumor immune responses in mice (49); −4, clinical trials of exosomes (50, 51); −5, discovery of exosomal RNA and transfer (12); −6, exosomes as a source of diagnostic biomarkers (52); −7, targeted delivery of siRNA through exosomes to brain (14).

Exosome-based drug delivery holds immense promise in the field of therapeutics including delivery of drugs across blood brain barrier and in the use of patient-derived microvesicles as a source of personalised drug delivery vehicle (13). The first proof-of-concept for the potential of exploiting these bioactive vesicles for targeted drug delivery was performed using dendritic cell-derived exosomes for siRNA delivery to the brain after systemic injection (14). The specificity of using exosomes as a drug carrying vehicles has created new opportunities for treatments of many diseases, most importantly, without significant side effects (15).

With the exponential increase in exosomal studies, the datasets generated are multidimensional originating from heterogeneous experimental platforms. Whilst most of the generated molecular (protein/RNA/lipid) data are mentioned in the inline text of the published article, a vast majority is often placed as supplementary information or not provided (especially with high throughput techniques) (16, 17). Importantly, whether in inline text or in supplementary tables, these exosomal molecular data in published articles are not easily queriable (16). In order to obtain novel biological insights, it is a perquisite to collate exosomal molecules in a centralized repository (18). For this reason, ExoCarta was created in 2009 as a free web-based resource that catalogs proteins and RNA identified in exosomes (19). ExoCarta is manually curated by expert scientists (http://www.exocarta.org) and contains molecular data on published and unpublished exosomal studies. It catalogs information on the exosomal isolation and purification procedures, samples used, investigator details and exosomal molecular components such as proteins, mRNA and miRNA as reported in the specific articles (20). ExoCarta can be queried using the gene symbol/name or browsed as a group based on the organism, molecular content type and the sample material. It is updated on a quarterly basis wherein newly published datasets are manually curated and appended to the existing repertoire of molecular data. Additionally, new features are added as per the demand of the exosomal field (for example, the importance of lipid molecules in exosomes prompted their inclusion in ExoCarta) (20).

## Utility of ExoCarta

**Fig. 2:**
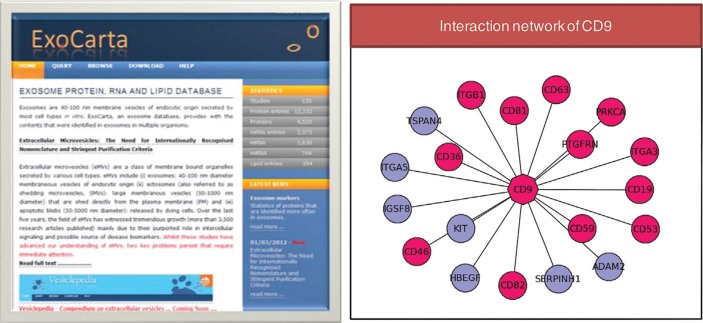
A snapshot of ExoCarta and interaction network of CD9. Snapshot of ExoCarta homepage is displayed. Protein interaction network of CD9 (tetraspanin family) shows its protein interactors that are identified in exosomes (pink) and not (blue). Each individual tetraspanin protein performs differently via actions through their respective interactors; for instance, CD9 and CD82 mediate metastasis inhibition by several mechanisms whereas CD151 supports tumour progression by activating MMPs (53, 54).

The current data and usage statistics of ExoCarta for the last 3 years are shown in Tables I and II, respectively. Currently, ExoCarta contains 12,232 and 3,139 protein and RNA entries, respectively (Table I). Few investigators have volunteered to submit their data directly to ExoCarta (http://www.exocarta.org/credits). In the following sections, the utility of ExoCarta is discussed:

- As shown in Table II, just over 16,000 unique users have visited ExoCarta over the last three years since its launch in 2009. The numbers are approximate and are based on unique IP addresses. A single user accessing ExoCarta from multiple computers will be counted multiple times. Also if multiple users from one educational institution access the database, they will be counted once. Regardless of the numbers, ExoCarta is a useful resource to the biological community.
- ExoCarta has been routinely used by various groups for establishing exosomal markers (21–28).
- Using the data downloaded from ExoCarta, 19 proteomic studies that identified at least 30 proteins were analysed to obtain the general protein composition of exosomes (9). Conversely, in addition to the conserved set of proteins, exosomes are also shown to contain a tissue-specific signature (11). The presence of tissue-specific signatures makes exosomes important in intercellular signalling and as possible source of disease biomarkers. Users can browse mRNA- and protein-based quantitative data to assess the tissue specificity of proteins and its presence in exosomes can be investigated through ExoCarta.
- In ExoCarta, the isolation procedures along with the buoyant density are listed where available. ExoCarta allows researchers to download the entire dataset and filter them based on specific methods (e.g. sucrose density gradient centrifugation) for further analysis. Gyorgy et al. performed meta-analysis on the datasets downloaded from ExoCarta filtered based on density gradient centrifugation (29). The study observed the enrichment in the number of membrane proteins and depletion in nuclear proteins in exosomes.
- Welton et al. performed a statistical over-representation analysis with datasets (limited to mass spectrometry) obtained from ExoCarta (30). The analysis highlighted a significant over-representation of proteins that are implicated in oncogenesis.
- Prior to the release of ExoCarta, investigators spend considerable amount of time to collate previous exosomal studies and comparing them with protein/RNA/lipid identifications from their own studies. ExoCarta now allows researchers to download all studies as a single file and thereby aid in a quick comparison. The use of ExoCarta in reducing erroneous protein identifications from datasets obtained from plasma and urine (31) and in aiding quick comparison has been acknowledged (31–36).
- From our preliminary analysis (Mathivanan and Simpson, unpublished observations), there is more than 60% overlap between proteins that are predicted to be non-classically secreted [based on SecretomeP (37)] and those detected in exosomes. Gyorgy et al. reported that 30% of cytoplasmic proteins identified in exosomes were predicted to be secreted by non-classical secretory pathways [SecretomeP (37)]. ExoCarta has been utilized to check for the presence of intracellular proteins that are detected in ovarian cancer ascites fluid (38) and wasp venom (39). Exosomal protein secretion as a possible mechanism of non-classical secretory pathway has prompted various groups to query ExoCarta for proteins lacking signal peptides (40, 41). Researchers can query ExoCarta to check whether their protein (not secreted by the classical secretory pathway) of interest is detected in any exosomal studies. Such additional information will prompt the investigators to design new studies to unravel the biological implications of exosome-based secretion.
- Koppen et al. used ExoCarta to identify orthologs of exosomal markers to characterize exosomes secreted by drosophila cells (42).
- It has been reported that immunoaffinity capture yields high quality exosomes, provided antibodies exist for the exosomal membrane protein of choice (43). Epithelial cell adhesion molecule (EPCAM), glycoprotein A33 (GPA33), HER2 (ERBB2) and CD63 molecule (CD63) are some of the previously used proteins to isolate exosomes from cell culture media or bodily fluids. As ExoCarta catalogs exosomal protein data from a wide range of cell types and tissues, it allows the investigators to choose the membrane protein that can be used for the exosomal immunoaffinity capture in their sample of interest. For example, GPA33 is not expressed in neuronal cells, which preclude it from being used for isolating exosomes that are of neuronal origin. However, neuronal membrane protein L1 cell adhesion molecule (L1CAM) or common proteins found in exosomes (CD63) can be used for isolating exosomes to homogeneity.
- As more functional roles of exosomes are uncovered, the implications of exosomal membrane and luminal proteins in signalling cannot be ignored. ExoCarta provides users with protein interactors for their molecule of interest [the protein-protein interaction data is obtained from Biological General Repositories for Interaction Datasets (BioGRID) (44) and Human Protein Reference Database (HPRD) (45)]. In the graphical display of protein interaction network present in the molecule page of ExoCarta, proteins detected in exosomes are highlighted in pink (Fig. 2). It can be speculated that, after the non-selective transfer of the luminal contents to the target cells, the transferred luminal proteins may interact with target cell proteins triggering downstream responses. Even though the molecular interactors vary depending on the cell type and the function performed, ExoCarta can provide information on the known protein interactors of the exosomal protein of interest (Fig. 2).

**Table I:** ExoCarta data statistics

1	Number of exosome studies	135
2	Number of protein entries	12,232
3	Number of proteins	4,520
4	Number of mRNA entries	2,375
5	Number of mRNA molecules	1,639
6	Number of lipid molecules	194
7	Number of miRNA molecules	764
8	Number of cell types from which exosomes were isolated	94
9	Number of body fluids from which exosomes were isolated	12

**Table II:** ExoCarta usage statistics

1	Number of unique visitors (July 2009–March 2012)	16,076
2	Number of unique visitors (average per month)	1,783
3	Number of page views (July 2009–March 2012)	324,310
4	Number of pages per visit	6.82
5	Average time on site	5.10 minutes

## The need for the involvement of exosomal community

With the explosion of exosomal studies, to maintain and update ExoCarta is a difficult task. However, with the active participation of the exosomal community, the database can be updated regularly. To facilitate active involvement of the exosomal research community, data contributions are acknowledged in the credits section of ExoCarta. The submitted datasets can be highlighted as private (only be accessed by the authors or their collaborators) or public (accessible by everyone).

## ExoCarta is not a gold standard

Isolation and purification protocols employed in any exosomal study are of paramount interest as high quality exosomes are needed for any biological study. As data compiled in ExoCarta are derived directly from published articles, the quality of the datasets in ExoCarta is as good as the published articles. *We emphasise caution when using datasets from ExoCarta as many published studies have purified exosomes by the far simpler differential centrifugation approach often ending with a crude preparation of exosomes with contaminants including ectosomes, apoptotic blebs and protein aggregates* (30, 43, 46). Whilst the exosomal community is addressing this issue, it has to be accepted that much of the published information has been obtained from impure preparations.

## Future directions

A manually curated compendium of extracellular vesicles (apoptotic blebs, exosomes, large dense core vesicles, microparticles, microvesicles and synaptic vesicles) called Vesiclepedia has recently been completed and will be launched soon. ExoCarta will be continuously maintained after the release of Vesiclepedia and will become a primary resource with high quality exosomal datasets. The quality control is critical, and inputs from the exosomal community through International Society of Extracellular Vesicles (ISEV) will aid in filtering the existing data and in the addition of new high quality datasets. One of the current problems with extracellular vesicles is the nomenclature used in naming the vesicles. The confusion in the nomenclature has led to typical exosome preparations sometimes being referred to as microvesicles and vice versa (46). With the involvement of the ISEV, the nomenclature of vesicles can be standardised and emphasis can be made on employing stringent purification protocols to isolate exosomes.
